# Algal Lectin Griffithsin Inhibits Ebola Virus Infection

**DOI:** 10.3390/molecules30040892

**Published:** 2025-02-14

**Authors:** Leah Liu Wang, Kendra Alfson, Brett Eaton, Marc E. Mattix, Yenny Goez-Gazi, Michael R. Holbrook, Ricardo Carrion, Shi-Hua Xiang

**Affiliations:** 1 Nebraska Center for Virology, School of Veterinary Medicine and Biomedical Sciences, University of Nebraska-Lincoln, Lincoln, NE 68588, USA; leahwang517@huskers.unl.edu; 2 Texas Biomedical Research Institute, 8715 W. Military Drive, San Antonio, TX 78227, USAygoez@txbiomed.org (Y.G.-G.);; 3 Integrated Research Facility at Fort Detrick, National Institute of Allergy and Infectious Diseases, Frederick, MD 21702, USA; 4 Nonclinical Pathology Services, LLC, 5920 Clubhouse Pointe Dr., Medina 44256, OH, USA

**Keywords:** griffithsin (GRFT), algal lectin, Ebola virus (EBOV), Ebola virus disease (EVD), mannose-binding, carbohydrate-binding

## Abstract

Algal lectin Griffithsin (GRFT) is a well-known mannose-binding protein which has broad-spectrum antiviral activity against several important infectious viruses including HIV, HCV, and SARS-CoV-2. Therefore, GRFT has been brought great attention to antiviral therapeutic development. In this report, we have tested GRFT’s activity against the lethal Ebola virus *in vitro* and *in vivo*. Our data have shown that the IC_50_ value is about 42 nM for inhibiting Zaire Ebola virus (EBOV) infection *in vitro.* The preliminary *in vivo* mice model using mouse-adapted EBOV has also shown a certain efficacy for delayed mortality compared to the control animals. A GRFT pull-down experiment using viral particles demonstrates that GRFT can bind to N-glycans of EBOV. Thus, it can be concluded that GRFT, through binding to viral glycans, may block Ebola virus infection and has potential for the treatment of Ebola virus disease (EVD).

## 1. Introduction

Griffithsin (GRFT) is a mannose-binding lectin which was isolated from the red alga *Griffithsia* sp. for anti-HIV study in 2005 [1]. GRFT has been shown to have strong antiviral activities against various important viruses such as HIV, HCV, and SARS-CoV-2 [2], and it has been used specifically in clinical trials as a microbicide for HIV infection control [3,4,5]. GRFT is a small protein with 121 amino acids, and the structure of GRFT is a typical Jacalin-like lectin fold domain which forms a homodimer [6,7,8]. GRFT mainly binds mannoses, which are the common sugars present on the surfaces of enveloped viruses [2,8]. Therefore, GRFT is able to neutralize enveloped viruses through binding to those mannoses on the viral surface and interfering with viral entry.

*Ebolavirus* is a genus of the family *Filoviridae* that can cause severe hemorrhagic fever, referred to as Ebola virus disease (EVD). There are six distinct Ebola viral species which have been reported: *Zaire ebolavirus* (EBOV), *Sudan ebolavirus* (SUDV), *Bundibugyo ebolavirus* (BDBV), *Tai Forest ebolavirus* (TAFV), *Bombali ebolavirus* (BOMV) and *Reston ebolavirus* (RESTV) [9,10]. The prototypical Zaire Ebola virus (EBOV) was first discovered in 1976, and has caused a number of deadly EVD epidemics in Africa with an average mortality of 50%. For this EVD, we only have a vaccine for limited use [11], and two antibody-based drugs which were approved in 2020 [12]. Therefore, it is essential to develop more effective therapeutics for treating this fatal infectious disease. Ebola viruses are enveloped, single-stranded negative-sense RNA viruses with a genome size of about 19 kb which encodes seven proteins: nucleoprotein (NP), viral protein 35 (VP35), VP40, glycoprotein (GP), VP30, VP24, and RNA polymerase (L) [13,14]. Glycoprotein (GP) is densely glycosylated with N-linked glycans (about 17 glycosylation sites on average) such as mannoses [15,16]. Consequently, GRFT can bind the glycans on the GP of an EBOV virion surface to interfere with GP interaction with viral cell receptors for viral entry. Like EBOV-antibody-based drugs [17], GRFT also targets EBOV GP and blocks viral entry, which implies that GRFT-based inhibitors will be promising for the development of anti-EBOV drugs [18].

In this report, we have demonstrated that GRFT has strong Ebola virus infection-inhibiting activities *in vitro* and *in vivo*. Our data suggest that GRFT will have the potential for therapeutic use to treat Ebola virus infection.

## 2. Results

### 2.1. GRFT Inhibits Ebola Virus Infection In Vitro

**Figure 1:**
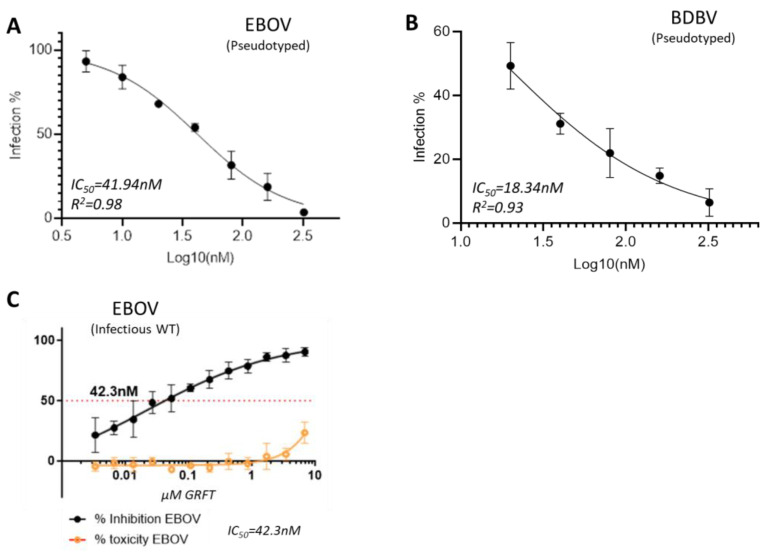
GRFT inhibition assays *in vitro*. Inhibition assay against Pseudovirus EBOV (**A**) and BDBV (**B**). Inhibition assay against infectious virus EBOV, which was conducted in BSL-4 containment (**C**). IC_50_, 50% inhibition concentration. All samples were tested in triplicate.

To know whether GRFT can inhibit the deadly Ebola virus infection, we first tested it in a pseudotyped virus-based platform which was established in our BSL-2 laboratory. Two Ebola viruses (EBOV and BDBV) were pseudotyped by using an HIV-1 backbone (pSG3ΔEnv), and were evaluated using TZM-bl cells by measuring the Luciferase activity. The results indicated that GRFT exhibited strong activity against both pseudotyped Ebola viruses, EBOV and BDBV, with IC_50_ values of 41.84 nM and 18.34 nM, respectively (Figure 1A,B). To validate the results, we then tested GRFT against authentic infectious wild-type Zaire Ebola virus (EBOV) in the BSL-4 containment (Makona strain at Fort Detrick, MD). The result was surprisingly comparable with the pseudovirus-based result, with an IC_50_ value of 42.3nM (Figure 1C). Hence, the belief that GRFT indeed inhibits Ebola virus infection with high potencies is well justified.

### 2.2. GRFT Delayed Mortality In Vivo

**Figure 2:**
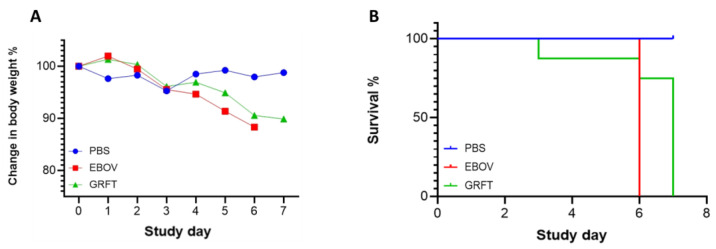
GRFT inhibition evaluation in mice. Balb/c mice were used in three groups of eight animals. One group was mock challenged with PBS and mock treated with vehicle. Two groups (EBOV and GRFT) were challenged with 1000 PFU of mouse-adapted EBOV by intraperitoneal injection. The EBOV-only group was treated with vehicle, and the GRFT group was treated with GRFT via a subcutaneous route twice per day. Body weight curves (**A**) and survival rates (**B**). The statistical analysis of survival rate was carried out via the Log-rank (Mantel–Cox) program in Prism, with significant difference shown with a *p* value of 0.0141 between vehicle-treated (red line) and GRFT-treated (green line) mice.

To further evaluate whether GRFT can inhibit Ebola virus infection *in vivo*, we applied the mouse model, by challenge, with a mouse-adapted EBOV strain (Kikwit isolate at Texas Biomedical Research Institute, San Antonio) in an animal-based ABSL-4 containment. Three groups (eight mice/group) were included (PBS mock, GRFT treated and untreated). The animals were infected by an intraperitoneal (IP) injection of 1000 plaque forming units (pfu) of EBOV. GRFT-treated animals were subcutaneously (SC) injected with GRFT (10 mg/kg) twice a day for 6 days. The data showed all untreated group animals died on day 6, but all GRFT treated group animals died on or by day 7 except one (Figure 2A,B). This indicates that GRFT delayed animals’ mortality by one day, although one animal died on day 3 from an unknown reason. In addition, EBOV-related histopathological findings were similar in character to those noted in the positive control group but were, in general, less frequent and in lower severity grades (see the details in the Pathology report in the Supplementary Data).

### 2.3. GRFT Binds to N-Glycans of EBOV-GP

**Figure 3:**
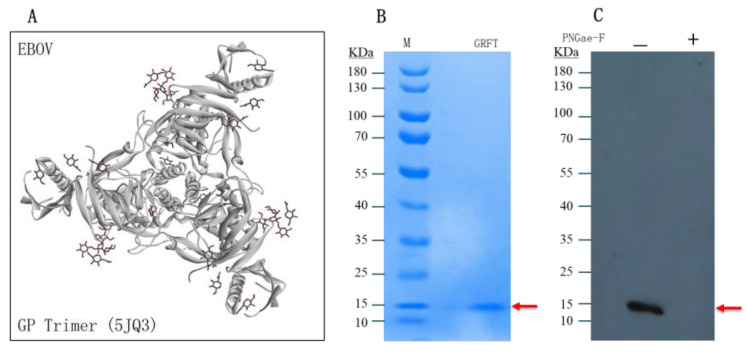
GRFT binding assay. (**A**) Glycans on the EBOV-GP trimers (~54 glycans/each trimer). (**B**) GRFT protein presence in the Coomassie blue gel, the size is about 14.5 KD (pointed by the red arrow). (**C**) Western blot showing GRFT was pull-down by EBOV particles, the GRFT band appeared by anti-His tag antibody as GRFT protein was tagged by 6xHis. If treated with PNGase-F, GRFT did not pull-down by EBOV.

To test whether the algal lectin GRFT binds N-glycans of EBOV-GP for antiviral function, we designed a binding assay called Virus pull-down using pseudotyped EBOV particles. If GRFT molecules can bind the N-glycans of viral particles, they will be precipitated (pull-down) with the viral particlesby centrifugation, since there are about ~54 glycans on an EBOV-GP-trimer (Figure 3A, PDB 5JQ3). The PNGase-F-treated EBOV particles, used to remove the glycans, were used as the negative control. Then, the treated and untreated EBOV samples were incubated with GRFT protein molecules for the binding reaction. These viral particles were precipitated by centrifugation. The pellets of virus particles were analyzed by Western blotting using anti-His antibody, as the GRFT protein molecules contain His-tags. In Figure 3, Western blot (Figure 3C) clearly shows that the pull-down GRFT protein band, with the corrected size of 14.5 KD, is matched to the size of the GRFT protein band stained with Coomassie blue, but the PNGase-F-treated sample obviously lacked this GRFT protein band (Figure 3B). These data strongly demonstrated that GRFT can bind the GP-trimers of EBOV particles. Thus, the bound GRFT will interfere with the virus–receptor interaction involved in viral entry.

### 2.4. GRFT Homologues Analysis

**Figure 4:**
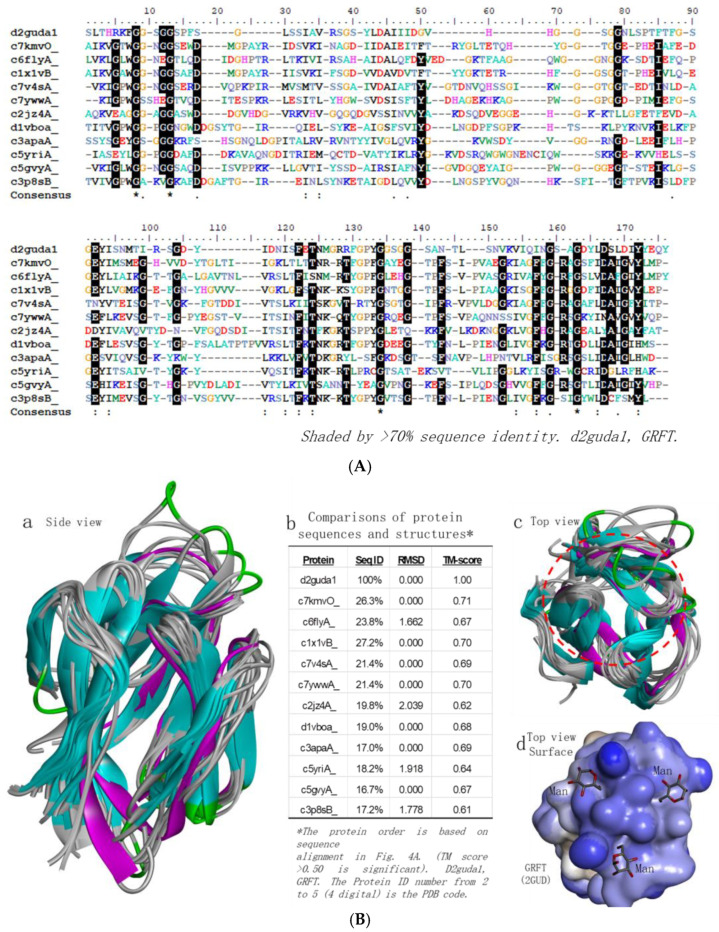
GRFT homologs analysis. (**A**) Top twelve hits for GRFT homologous protein sequence alignment from a structure-based dataset search using the Phyre2 program [19]. The sequences that exhibit more than 70% homology are shaded in black, identical (*), similarity: lower (.), higher (:). The d2uda1 is GRFT. (**B**) Comparisons of sequences and structures from GRFT homologous proteins Sequence comparison (**b**), Superimposition of the top twelve homologous protein structures: side view (**a**), top view (**c**), and glycans (mannose, Man) binding model on the surface of GRFT (made from PDB 2GUO) (**d**).

To find out whether other proteins which may be similar to GRFT have the potential to bind EBOV and neutralize the viruses, we conducted GRFT structure-based searches in the protein databank (PDB) using Phyer2 [19]. The top twelve hits were analyzed and are presented in Figure 4. Interestingly, from their sequence alignment, they do not show significant homologous levels (only ~20% identities) (Figure 4A), but they showed significantly homologous levels with regard to their structures (Figure 4B). In the superposition of their structural models, they all have small RMSDs (root mean square deviations), in the range of 0.00 to 2.039, which suggests that they are highly homologous structurally. Their high TM scores, which are higher than 0.61, indicate that the comparison data are generated with high confidence (Figure 4B(b)). Interestingly, the top homologous proteins are plant-based proteins, such as banana (c7kmvO, c1x1vB) [20,21], pineapple (c6flyA) [22], barley (c7v4sA) [23] and rice (c2jz4A) [24]. Through the GRFT homology analysis, there are actually many GRFT homologues that are worthy of being explored for the sake of finding new antiviral agents.

## 3. Discussion

We have demonstrated that GRFT can neutralize Ebola virus at high potency through binding the glycans of viral particles. Certainly, GRFT is a broad-spectrum inhibitor against several major enveloped viruses, such as HIV, HCV, HPV, SARS-CoV-2, and Ebola virus. It can be assumed that GRFT would have activity against other filoviruses, such as Marburg virus (MARV). The genomic sequence of MARV has about 54% identity with EBOV, and MARV-GP is also highly glycosylated [15,16].

GRFT is safe for therapeutic applications [2,18]. It has been used as a microbicidal agent against HIV infection [5], and also been tested for *in vivo* use by injection, such as demonstrated in murine models (mice and guinea pigs), where it has been seen that GRFT is safe even in the case of high-dose injection [25,26]. GRFT has not been found to cause robust immune responses in T-cells, but rather a lack of T-cell immune response [27], and, additionally, no antibody induction being detected [25]. For the prevention of Ebola virus infection, the treatment window is only a few days; the antibody response will take weeks, so the efficacy will not be affected by immune responses. Therefore, GRFT has been recognized as the best lectin candidate for therapeutic use [2,16,28]. A recent report has showed that GRFT has protected Syrian golden hamsters from infection with the lethal Nipah virus [29]. There have also been reports of *in vivo* GRFT (mice) activity reducing viral titers in HCV infection [30], SARS coronavirus (SARS-CoV) infection [31], and Japanese encephalitis virus (JEV) infection [32]. Other lectins were reported previously to have activities against filovirus infections, such as banana lectin (BanLec) [33,34], Cyanovirin-N [33], and Scytovirn [35,36]. In this report, we also attempted *in vivo* studies on mice using subcutaneous (SC) injection. The preliminary experiment showed a limited effect, as only one dose was used, which is clearly a low dose, but we can still see some protection efficacy. Although no animals survived, GRFT delayed the occurrence of mortality (see Figure 2 and Supplementary Materials File S1). It is suggested that GRFT played a certain role against viral infection. Nevertheless, more optimized tests in animal models are required for developing the therapeutic use of GRFT. More importantly, nonhuman animal models are also needed for testing, to more closely represent humans. In addition, GRFT can be used for treatment in combination with other drugs to achieve synergistic effects. For example, it may be combined with an RNA-dependent RNA polymerase (RdRp) inhibitor (i.e., Remdesivir); since they have different targets, a synergistic effect will be produced. There have also been reports on increasing the production of GRFT, such as from plants or cell-free systems [37,38]. Therefore, high yield production has provided assurance of commercialization for therapeutic applications.

Finally, based on the GRFT-structure-based search, there are lots of GRFT-homologous carbohydrate-binding proteins (CBP), which may be a rich source for finding new antiviral agents.

In conclusion, GRFT has been demonstrated to have strong activity against the deadly Ebola viruses through binding to the N-glycans of viral particles. It has potential for therapeutic development for the treatment of Ebola virus disease.

## 4. Materials and Methods

### 4.1. Viruses, Plasmids, and Cells

The envelope glycoprotein genes (GPs) synthesized are based on the sequences from GenBank for Ebola virus (Zaire ebolavirus, accession number: AIO11753.1) and Bundibugyo ebolavirus (GenBank accession number: AGL73460). The plasmid pSG3ΔEnv, was from the NIH AIDS Reagent Program. Griffithsin (GRFT), TZM-bl, and 293T cells were requested from the NIH AIDS Reagent Program. Ebola virus strain Makona C07 (IRF0192) and Huh7 cells were used for inhibition assays in the BSL-4 containment in the NIH Integrated Research Facility at Fort Detrick.

### 4.2. Pseudotyping Viruses

All pseudotyped viruses were made from HIV-1 backbone plasmid pSG3ΔEnv, as effectively demonstrated by the HIV-based, pseudotyped Ebola [39]. The envelope genes (GPs) of the Ebola viruses were synthesized and cloned into the pCDNA3.1+ expression plasmid. Both plasmids of pSG3ΔEnv and the GP envelope were co-transfected into 293T cells in a 10 cm plate using transfection reagent polyethyleneimine (PEI). Incubation was carried out at 37 °C for two days, and the supernatants were harvested after a short spin to remove cell debris and stored at −80 °C.

### 4.3. Inhibition Assay Against Pseudoviruses

Virus neutralization assay was performed in the BSL-2 laboratory in 96-well plates using pseudotyped Ebola viruses and TZM-bl cells (6000/well) as this cell-line was engineered with a Luciferase report gene under the inducible promoter of Tat factor. The viral particles and peptide samples were mixed and transferred onto the target cell wells for infection. One-day post-infection, the supernatants were removed, the cells were washed once with PBS, and they were incubated in fresh media for one more day. Then, the cells were lysed in 1X Passive Lysis Buffer (Promega, Madison, WI, USA) and kept at room temperature for 20 min for luciferase assay. Luciferase activity was measured using luciferin substrate (Promega) in the Veritas Luminometer. Neutralization activity was calculated by comparison with control samples.

### 4.4. Inhibition Assay Against Infectious Ebola Virus

Huh7 cells were seeded with 6000 per well in 30 µL using a 384-well plate, with cells being allowed to grow for 24 h. Serial diluted compound (Griffithsin) solutions were mixed with viruses (Ebola virus strain Makona C07 at Fort Detrick, MD, USA) at a total volume of 20 µL and incubated for 1h, and then were loaded onto cells and incubated for 48 h–72 h. At least 50 µL of 20% formalin was added to each well using Viaflo 384, and the product was let stand for 30 min. Plates were then removed from biocontainment IAW, following standard operating procedures. The plates were stained with fluorescent probes and imaged using a Perkin-Elmer Operatta Automated Microscope (Waltham, MA, USA). The data were analyzed in GraphPad Prism (https://www.graphpad.com/) [40].

### 4.5. Mice Model Study

Twenty-four Bal/c mice (7 weeks of age) in three groups (PBS, EBOV only, and EBOV-GRFT-treated) were tested. Mouse-adapted viruses (1000 pfu) were administered by intraperitoneal (IP) injection to the two EBOV groups; the mock control PBS group was challenged with PBS only, and mock treated with vehicle only. Compound (griffithsin, GRFT) treatment was administered by subcutaneous (SC) injection of ~1.0 mg/kg (8 µg/dose), twice a day. Animals were monitored and weighed daily. When moribund (or at a scheduled end-of-study, on day 21 post-challenge for the mock infection group), animals were euthanized with CO_2_, and blood and tissue samples (liver, spleen, lung) were taken for viral load and histopathology analysis [41,42,43], and reporting (see Supplementary Materials File S1). All animal experiments conducted strictly followed the IACUC of the Texas Biomedical Research Institute approved protocols (TXBIO2018-007, IACUC #1648MU3), in compliance with the Animal Welfare Act PHS policy.

### 4.6. Virus Pull-Down Assay

Pseudoviruses (EBOV) (10 µL, 2,000 FLU units/µL) were incubated with PNase-F enzyme (5 µL, 500 units/µL, New England BioLabs, Ipswich, MA, USA) at 37 °C for 24 h. The PNase-F treated or untreated pseudovirus was mixed with griffithsin (GRFT) (5 µg) at a volume of 500 µL, and the product was incubated at room temperature for 1 h. Then, the two samples were centrifuged with 16,000× *g* at 4 °C for 2 h. After removing the supernatants, the virus pellets were treated with a gel loading buffer and boiled for 5 min before being loaded into the PAGE gel (12%). To detect the pull-down GRFT protein, a standard Western blot was performed using anti-His antibody, as the GRFT protein was tagged with 6xHis.

### 4.7. Molecular Modeling

Several programs were used for bioinformatics and structural analysis. BioEdit (version 7.2) and ClustalW (https://www.genome.jp/tools-bin/clustalw, accessed on 9 February 2025) for multiple-sequence alignment, Phyre2 (http://www.sbg.bio.ic.ac.uk/phyre2, accessed on 9 February 2025) [19] for structure-based search and Discovery Studio (Visualizer) (BIOVIA, https://discover.3ds.com/discovery-studio-visualizer, accessed on 9 February 2025) for structural modeling.

## Data Availability

The original contributions presented in this study are included in the article/Supplementary Materials. Further inquiries can be directed to the corresponding author.

## Supplementary Materials

The following supporting information can be downloaded at: https://www.mdpi.com/article/10.3390/molecules30040892/s1. File S1. Summary Pathology Report.
