# Supplementary material for: Algal Lectin Griffithsin Inhibits Ebola Virus Infection
Source: Molecules. 2025 Feb 14;30(4):892. doi: 10.3390/molecules30040892 (PMC11858388; doi:10.3390/molecules30040892)
Supplement: Supplementary file 1 [file molecules-30-00892-s001.zip › molecules-3386795-Supplementary file S1.pdf]

SUMMARY PATHOLOGY REPORT

1648 MU

(Data report showing here for GRFT only)

Efficacy of Novel Ebola virus Countermeasures in Mice

Study Director: Ricardo Carrion Jr., PhD
Texas Biomedical Research Institute

Study Pathologist: Marc E. Mattix, DVM, MSS
Diplomate, ACVP
Nonclinical Pathology Services, LLC

COMPILED FOR:
Texas Biomedical Research Institute
8715 W. Military Dr.
San Antonio, TX 78227-5302

TABLE OF CONTENTS

**INTRODUCTION**	**Page**	**3**
**Study Title**		**3**
**Purpose**		**3**
**MATERIALS AND METHODS**		**3**
**PATHOLOGY RESULTS**		**3-6**
**Survival**		**3**
**Histologic findings**		**4-6**
**Group 9**		**4**
**Group 10**		**4**
**Group 13**		**6**
**CONCLUSION**		**7**
**REFERENCES**		**7**
**REPORT SUBMISSION**		**8**

INTRODUCTION

Study Title: Efficacy of novel Ebola virus countermeasures in mice.

Purpose: The purpose of this study is to evaluate the efficacy of novel EBOV countermeasures in mice.

MATERIALS AND METHODS

Study Site: Texas Biomedical Research Institute
Animals: Mice
Challenge Agent: Mouse adapted (MA) EBOV, 1000 PFU IP
Test Articles: KE-163922; KE-168824; GRFT
Vehicle: PBS

Experimental Study: Forty mice were assigned to 5 groups consisting of 8 mice per group, as detailed in Table 1.

Table 1. Experimental Design *(Only showing for GRFT)*

Group No.	No. Animals	Experimjental Objective	Treatment SC	Challenge Agent
9	8	PBS Mock; vehicle treated, unexposed	N/A	N/A
10	8	Vehicle treated, MA EBOV exposed	N/A	MA EBOV
13	8	GRFT SC	SD 0,9 (SID)<br>SD1-8 (BID)	MA EBOV

N/A – Not applicable.

Gross Necropsy:
Necropsies were conducted in accordance with Texas Biomed SOP 903. Tissue samples (liver, spleen, and lung) were collected for microscopic evaluation.

Histopathology:
Tissues were fixed by immersion in 10% neutral-buffered formalin for a minimum of fourteen days, then trimmed, routinely processed, and embedded in paraffin. Sections of the paraffin-embedded tissues were cut at 5 µm thick, and histology slides were deparaffinized, stained with hematoxylin and eosin (H&E), coverslipped, and labeled. Slides were evaluated by a board-certified veterinary pathologist using a light microscope. The results of histopathological examination of each animal were summarized in the Summary Pathology Report by the Study Pathologist (Table 2 to Table 6).

PATHOLOGY RESULTS

Survival
All PBS unexposed control animals survived to the Day 21 terminal euthanasia. Two of 8 animals assigned to the KE-168824 group (Group 12) survived to the terminal euthanasia. All vehicle treated MA EBOV exposed mice (Group 2) and remaining test article-treated MA EBOV-exposed mice (Group 11, 12, and 13) died or were euthanized prior to the end of protocol, between Day 3 and 7.
GROUP 13 (GRFT)

<u>Histologic findings</u>

Microscopic changes consistent with EBOV infection consisted of hepatocellular necrosis with mixed cell inflammation and intracytoplasmic inclusion bodies and splenic lymphoid depletion with lymphocytolysis. EBOV-related findings were similar in character to those noted in the positive control and other treated groups but were in general less frequent and in lower severity grades. One finding noted within the liver, hepatocellular vacuolation, was not noted in the control groups. The finding was characterized by enlarged hepatocytes that contained numerous small clear cytoplasmic vacuoles with discrete, well-defined margins (microvesicular vacuolation). Findings are summarized in Table 6.

Table 6. Summary of select Group 13 histopathology findings

	Accession No.	24-0104							
	Study Day	7	7	3	7	6	7	7	7
	Animal No.	A	B	C	D	E	F	G	H
LIVER	Hepatocellular necrosis	0	1	0	3	1	1	2	3
	Inflammation	0	0	0	1	1	1	1	2
	Hepatocellular vacuolation	0	0	0	2	0	0	2	1
	Cytoplasmic inclusions	0	0	0	P	P	0	0	0
	Mononuclear cell infiltration	0	0	0	0	0	0	0	0
SPLEEN	Lymphoid depletion	0	1	0	1	0	0	1	0
	Lymphocytolysis	0	1	0	1	0	0	1	0
	Fibrin	0	0	0	0	0	0	0	0
	Necrosis, focal	0	0	0	0	0	0	1	0
	Lymphoid hyperplasia	0	0	0	0	0	0	0	0
LUNG	Inflammation	0	0	0	0	0	0	0	0
	Increased alveolar macrophages	0	0	0	0	0	0	0	0

1- Minimal; 2- Mild; 3- Moderate; 4- Marked; 5- Severe; P - Ungraded finding present; 0 - Finding not present; NE - Not evaluated

CONCLUSION

All GRFT-treated mice died or were euthanized between Day 3 and 7. Of interest, EBOV-related microscopic changes were less frequently noted in this group and were generally of lower severity grade when compared with the other two treatment group mice. Two of the GRFT-treated mice lacked EBOV-related microscopic findings in the tissues evaluated.

REFERENCES

Bray M, Davis K, Geisbert T. A mouse model for evaluation of prophylaxis and therapy of Ebola hemorrhagic fever. J Infect Dis 1998;178:651-661.

Gibb TR, Bray M, Geisbert T, Steele KE, et al. Pathogenesis of experimental Ebola Zaire virus infection in BALB/C mice. J Comp Pathol 2001;125:233-242.

REPORT SUBMISSION

Texas Biomed 1648 MU Pathology Report

<u>Report Submitted By:</u>

Marc E. Mattix, DVM, MSS
Diplomate, ACVP
Study Pathologist
